# ZO-1 Intracellular Localization Organizes Immune Response in Non-Small Cell Lung Cancer

**DOI:** 10.3389/fcell.2021.749364

**Published:** 2021-12-06

**Authors:** Déborah Neyrinck-Leglantier, Julien Lesage, Silvia Blacher, Arnaud Bonnomet, Walter Hunziker, Agnès Noël, Valérian Dormoy, Béatrice Nawrocki-Raby, Christine Gilles, Myriam Polette

**Affiliations:** ^1^ University of Reims Champagne-Ardenne, Inserm UMR-S 1250, SFR CAP-Santé, Reims, France; ^2^ Department of Internal Medicine-Medical Oncology, Washington University, St. Louis, MO, United States; ^3^ Laboratory of Tumor and Development Biology, GIGA-Cancer, University of Liège, Liège, Belgium; ^4^ Cellular and Tissue Imaging Platform, University of Reims Champagne-Ardenne, Reims, France; ^5^ Epithelial Cell Biology Laboratory, Institute of Molecular and Cell Biology, Singapore, Singapore; ^6^ Department of Physiology, Yong Loo Lin School of Medicine, National University of Singapore, Singapore, Singapore; ^7^ Laboratory of Pathology, CHU of Reims, Reims, France

**Keywords:** epithelial plasticity, ZO-1, epithelial-mesenchymal transition, immune infiltrate, tumor microenvironment, lung cancer

## Abstract

Delocalization of zonula occludens-1 (ZO-1) from tight junctions plays a substantial role in epithelial cell plasticity observed during tumor progression. *In vitro*, we reported an impact of ZO-1 cyto-nuclear content in modulating the secretion of several pro-inflammatory chemokines. *In vivo*, we demonstrated that it promotes the recruitment of immune cells in mouse ear sponge assays. Examining lung cancers, we showed that a high density of CD8 cytotoxic T cells and Foxp3 immunosuppressive regulatory T cells in the tumor microenvironment correlated with a cyto-nuclear expression of ZO-1. Taken together, our results support that, by affecting tumor cell secretome, the cyto-nuclear ZO-1 pool may recruit immune cells, which could be permissive for tumor progression.

## Introduction

Epithelial cell plasticity, exemplified by Epithelial-Mesenchymal Transition (EMT), has crucial implications in tumorigenesis and metastasis (Dongre and Weinberg, 2019; Yang et al., 2020). A key event of EMT processes is the reorganization of intercellular junctions, particularly tight junctions (TJ). TJs are composed of transmembrane proteins (occludin, claudins, and junctional adhesion molecules) linked to the actin cytoskeleton through submembrane adaptor proteins including those of the zonula occludens (ZOs) family (González-Mariscal et al., 2020; Otani and Furuse, 2020). ZO-1, ZO-2, and ZO-3, belonging to the membrane-associated guanylate kinase homologs (MAGUK) family, are scaffold proteins involved in numerous protein-protein interactions through specific conserved domains: 3 PSD95/DLG/ZO-1 (PDZ) domains, one Src homology 3 (SH3) domain and one guanylate kinase homologous (GK) domain. Additionally, ZOs are also known as “shuttle” proteins harboring nuclear export signal (NES) and nuclear localization signal (NLS) sequences that enable their translocation from the membrane to the cytoplasmic-nuclear compartment. We and others previously demonstrated that a cytoplasmic/nuclear relocation of ZOs is associated with EMT and high infiltrative capacities (Reichert et al., 2000; Polette et al., 2007; Luczka et al., 2013; Kyuno et al., 2021).

ZO-1, the first described member of the ZOs, has generally been considered a tumor suppressor with a reported diminished expression for instance in breast or colorectal cancers (Kaihara et al., 2003; Martin et al., 2004). ZO-1 is however found overexpressed in different types of cancers including pancreatic, gastric or melanoma (Kleeff et al., 2001; Resnick et al., 2005; Smalley et al., 2005). Despite the influence of ZO-1 expression levels, data have accordingly accumulated revealing a dual role of ZO-1 depending on its subcellular localization (Gonzalez-Mariscal et al., 2007; Polette et al., 2007; Bauer et al., 2010; González-Mariscal et al., 2020). Using a growth factor-induced EMT model, we previously reported that, a diminution of membrane-associated ZO-1 and its cyto-nuclear relocation correlated with enhanced expression of the pro-inflammatory cytokine interleukine-8 (IL-8) in invasive breast cancer cells (Brysse et al., 2012). Strengthening these findings, we further reported that cyto-nuclear ZO-1 promoted angiogenesis through a ZO-1/NFκB/IL-8 axis (Lesage et al., 2017).

In the last decade, the impact of EMT processes in generating a pro-tumoral tumor microenvironment has been demonstrated, notably through a differential activation or recruitment within the tumor microenvironment of host cells including tumor-associated macrophages (TAMs), tumor-associated neutrophils (TANs), tumor-infiltrating lymphocytes (TILs), cancer-associated fibroblasts (CAFs) or endothelial cells. Thus, several studies identified an increased expression of pro-inflammatory and pro-angiogenic soluble factors such as tumor necrosis factor-alpha (TNF-α), transforming growth factor-beta (TGF-β), interleukins-6 and -8 (IL-6, IL-8), and vascular endothelial growth factor (VEGF) in EMT-positive cell microenvironment (Le Bitoux and Stamenkovic, 2008; Dominguez et al., 2017; Suarez-Carmona et al., 2017). In addition, Suarez-Carmona et al*.* have established a correlation between the presence of vimentin-positive tumor cells and a myeloid cell infiltrate in triple-negative breast cancers (Suarez-Carmona et al., 2015). Other studies have shown that monocyte chemoattractant protein-1 (MCP-1) and IL-8 are regulated *via* the β-catenin pathway, especially during EMT programs (Levy et al., 2002; Mestdagt et al., 2006; Dutta et al., 2018; Wen et al., 2020).

In this study, we examined the involvement of ZO-1 in modulating the inflammatory infiltrate into the non-small cell lung cancer (NSCLC) tumor microenvironment. We demonstrated that cyto-nuclear ZO-1 is involved in the establishment and development of an immune microenvironment that could be permissive for tumor invasion in lung cancer.

## Materials and Methods

### Cell Lines and Culture Conditions

Cell lines were obtained from the American Type Culture Collection (Manassas, VA, United States). All culture media and reagents were from Gibco (Invitrogen, Carlsbad, CA, United States). BEAS-2B human lung cells and SKBR3 human mammary cells were cultured in DMEM containing 10% fetal bovine serum and 1% penicillin-streptomycin. THP-1 monocytic human cells were grown in RPMI medium supplemented with 20% FCS, and 1% penicillin-streptomycin.

### Plasmid and cDNA Transient Transfection

The expression vector that encodes wild-type ZO-1 (ZO-1) has been previously described (Reichert et al., 2000). The plasmid was transfected using the X-tremeGENE nine DNA reagent (Roche Diagnostics, Mannheim, Germany) on 1 × 10^5^ cells plated in 6-well plates according to manufacturer instructions. Cells were harvested 48 h later for quantitative RT-PCR and western blotting analyses. Serum-free conditioned media were collected for *in vivo* sponge assay and cytokine array analyses.

### Cytokine Array

The Proteome Profiler Human XL Cytokine Array kit (ARY022B, R&D) was used according to the manufacturer’s instructions. Briefly, conditioned media from BEAS-2B cells transfected with the ZO-1 expression vector or the empty vector was diluted with a mixture of biotinylated antibodies. Then, the mix was incubated overnight on a nitrocellulose membrane on which capture antibodies for 102 soluble factors had been coated in duplicate. After washing steps and incubation with horseradish peroxidase-coupled streptavidin, chemiluminescent detection was performed using ECL Prime (Pierce). The intensity of each spot was measured using MultiGauge (V3.0; Fujifilm; Tokyo; Japan). Each spot corresponding to a chemokine was quantified and normalized with the intensity of positive and negative control spots on the membrane. Data are expressed as fold induction for each chemokine in the ZO-1 transfected condition versus the pLNCX control condition.

### Transmigration Assay

Transmigration assays were performed as previously described (Brysse et al., 2012). THP-1 cells (10^5^) were suspended in 200 µl of serum-free DMEM containing 0.5% bovine serum albumin (BSA) and placed in the upper compartment of a transwell (6.5-mm diameter with 8 µm pore polycarbonate membrane insert; Costar). The lower compartment was filled with 600 µl of 48 h conditioned serum-free DMEM medium of BEAS-2B cells transfected or not with ZO-1 cDNA expression vector. After 24 h of incubation at 37°C, migrated THP-1 cells in the lower chamber of the transwell were counted with an ADAM automated cell counter. Results are expressed as fold induction to the empty expression vector control condition. Each experiment was carried out at least 3 times.

### Sponge Assay, Immunohistochemistry and Quantification

Sponge assays were performed as previously described (Van de Velde et al., 2018) with the approval of the ethical committee of the University of Liège (Approval No. 1599). Briefly, gelfoam sponges (Pfizer, New York, United States) were soaked in conditioned medium. Sponges were then subcutaneously inserted into the ears of BALB/c mice (Charles Rivers, Chatillon-sur-Chalarone, France) for 3 weeks. The experiment was repeated 3 times with five mice per group. At the end of the experiments, ears were collected, fixed in formol, and paraffin-embedded. Five micron-thick sections were realized across the sponge before immunostaining with a rabbit monoclonal anti-CD3 antibody (1:500; CD3; SP7; Ab16669; Abcam). Slides were scanned using the VS120 OLYMPUS (Olympus France SAS, Rungis, France) at a ×20 magnification. Image processing and quantification of cell density were performed using the images analysis toolbox of MATLAB 2019/B, according to the following steps: original images were registered in the full-color red, green, blue where sponge and cells appear in blue and red respectively. Classical and morphological filters were applied to eliminate the noise and enhance the contrast between the cells and the sponge. Finally, images were binarized using an automatic thresholding technique (Kapur et al., 1985). From binarized images, cells density was defined as the number of pixels belonging to all cells (total area occupied by cells) divided by the number of pixels belonging to the associated mask (filled area of the considered region of the sponge). The density values of labeled cells relative to the sponge area in the ZO1 groups were normalized to the mean density value of the control group.

### Human Lung Tumor Cohort

Human tissue samples were obtained from 42 patients with NSCLC [21 adenocarcinomas (ADC) and 21 squamous cell carcinomas (SCC)] (Supplementary Table S1). The tumors were staged according to the eighth TNM UICC/AJCC edition. Clinical data such as age and gender were collected retrospectively. Access to patient data for this retrospective non-interventional study was approved by the French national commission CNIL (Comité National de l’Information et des Libertés) (NO.2049775 v0). Paraffin-embedded tumor samples were obtained from the Tumor Bank of Reims University Hospital Biological Ressource Collection (No. AC-2019-340) declared at the Ministry of Health according to the French Law, for use of tissue samples for research.

### Immunohistochemistry on Human Samples

Immunohistochemistry for ZO-1, CD3, CD4, CD8, and Foxp3 was performed on serial sections of the 42 paraffin-embedded NSCLC tumor samples. ZO-1 and Foxp3 detection were performed as previously described (Lesage et al., 2017). CD3, CD4 and CD8 detection was performed with a Ventana Benchmark XT (Roche diagnostics GmbH). Each stage of the experiment was performed automatically according to the manufacturer’s instructions and driven by Ventana Medical Systems software, BenchMark XT module IHC/ISH (Ventana Medical Systems, Roche). Antibodies used for immunohistochemistry are listed in the supplementary material (Supplementary Table S2). A blind evaluation of the labeling and scoring was performed by two independent pathologists. Membrane-associated ZO-1 (tumor areas displaying a honeycomb ZO-1 staining), and cyto-nuclear ZO-1 staining, were scored as follows: 0 = no detection, 1 = detection in <10% of tumor cells, 2 = detection in 10–25% of tumor cells, 3 = detection in 26–50% of tumor cells, 4 = detection in >50% of tumor cells. From this scoring, cancers were categorized using a cutoff at 10%, as previously reported (Lesage et al., 2017), into a group combining cancers with scores 0 and 1 (<10%) and a group combining cancers with scores 2 to 4 (≥10%). CD3, CD4, CD8, and Foxp3 staining were scored as follows: 0 = no detection, 1 = detection in <10% of cells, 2 = detection in 10–25% of cells, 3 = detection in 26–50% of cells, 4 = detection in >50% of cells. Cancers were grouped in two categories: “low” expression group = cancers with scores from 0 to 2, “high” expression group = cancers with scores from 3 to 4.

### Statistical Analysis

Statistical analyses were performed with Prism (GraphPad Software, La Jolla, CA, United States). *In vitro* results expressed as fold induction were analyzed using the two-tailed one-sample Student’s *t*-test. *In vivo* results were analyzed using the two-tailed non-parametric Mann Whitney test. For human immunohistochemistry, the association between ZO-1 and CD3, CD4, CD8, and Foxp3 expression in NSCLC was studied by using Fisher’s exact test. A value of *p < 0.05 was considered statistically significant.

## Results

### ZO-1 Promotes Inflammatory Chemokine Secretion *in Vitro*

**FIGURE 1:**
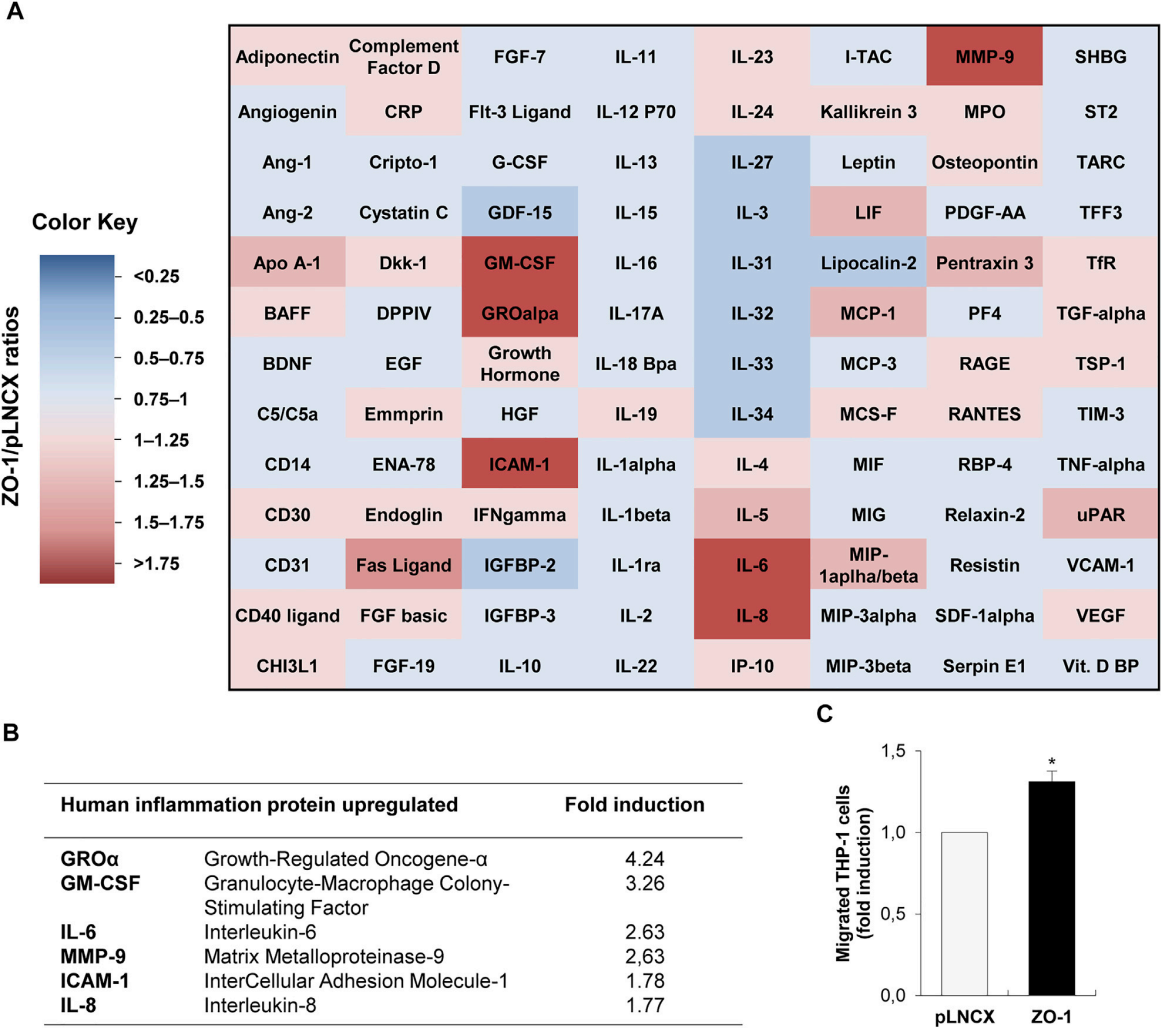
ZO-1 modulates *in vitro* inflammatory chemokine secretion. **(A)** Cytokine and chemokine secretion analyzed by cytokine-array in conditioned medium from BEAS-2B cells transfected with ZO-1 expression vector (ZO-1) or the corresponding pLNCX control vector (pLNCX). The heat map colors correspond to the pLNCX / ZO-1 cytokine and chemokine ratios. Downregulated (blue) and upregulated (red) proteins in conditioned medium from ZO-1 cDNA BEAS-2B cells are represented. **(B)** Summary table of most modulated chemokines according to the analysis of the cytokine/chemokine array presented in **(A)**. **(C)** Analysis of chemotactic migration of THP-1 monocytic cell in response to conditioned medium from ZO-1 cDNA BEAS-2B cells (conditioned medium of pLNCX empty vector transfectants is used as control). Data are expressed as fold induction relative to the control condition in three independent experiments. Mean ± SEM; *n* = 3; **p < 0.01.

Aiming to decipher a potential functional role of cyto-nuclear ZO-1 on inflammatory cell recruitment, we transiently transfected BEAS-2B invasive lung cells, which express low levels of ZO-1 maintained as a cyto-nuclear pool (Lesage et al., 2017), with ZO-1 cDNA expression vector or the empty pLNCX control vector. As previously reported in this cell model, we observed an increase of the cyto-nuclear pool of ZO-1 in BEAS-2B cells transfected with the ZO-1 expression vector compared to controls cells (Lesage et al., 2017). To validate our hypothesis that cyto-nuclear ZO-1 may modulate the secretome of tumor cells and thereby control the chemotactic activity of tumor cells, we examined the expression of 104 human soluble factors by cytokine array in conditioned medium of cells transfected or not with ZO-1. The modulation of the secretome by ZO-1 was characterized by at least a 50% increase in the production of cytokines, while no cytokine was found decreased below 50% (Figure 1A). The six most over-produced pro-inflammatory molecules were growth-regulated oncogene-α (GROα), granulocyte-macrophage colony-stimulating factor (GM-CSF), intercellular adhesion molecule-1 (ICAM-1), IL-6, IL-8, and matrix metalloproteinase-9 (MMP-9) (Figures 1A,B; Supplementary Figure S1). As a proof of concept, we used a transmigration assay with the THP-1 monocytic cell line to assess the chemotactic activity of tumor cells modified as above for cyto-nuclear ZO-1 content. Our results revealed that THP-1 cells migrated 30% more in response to conditioned media from ZO-1 cDNA-transfected cells than to conditioned media from control cells (Figure 1C). These results together demonstrate that tumor cells expressing high levels of cyto-nuclear ZO-1 secrete soluble factors that can stimulate inflammatory cell migration *in vitro*.

### ZO-1 Induces Immune Response Into Tumor Microenvironment *in Vivo*

**FIGURE 2:**
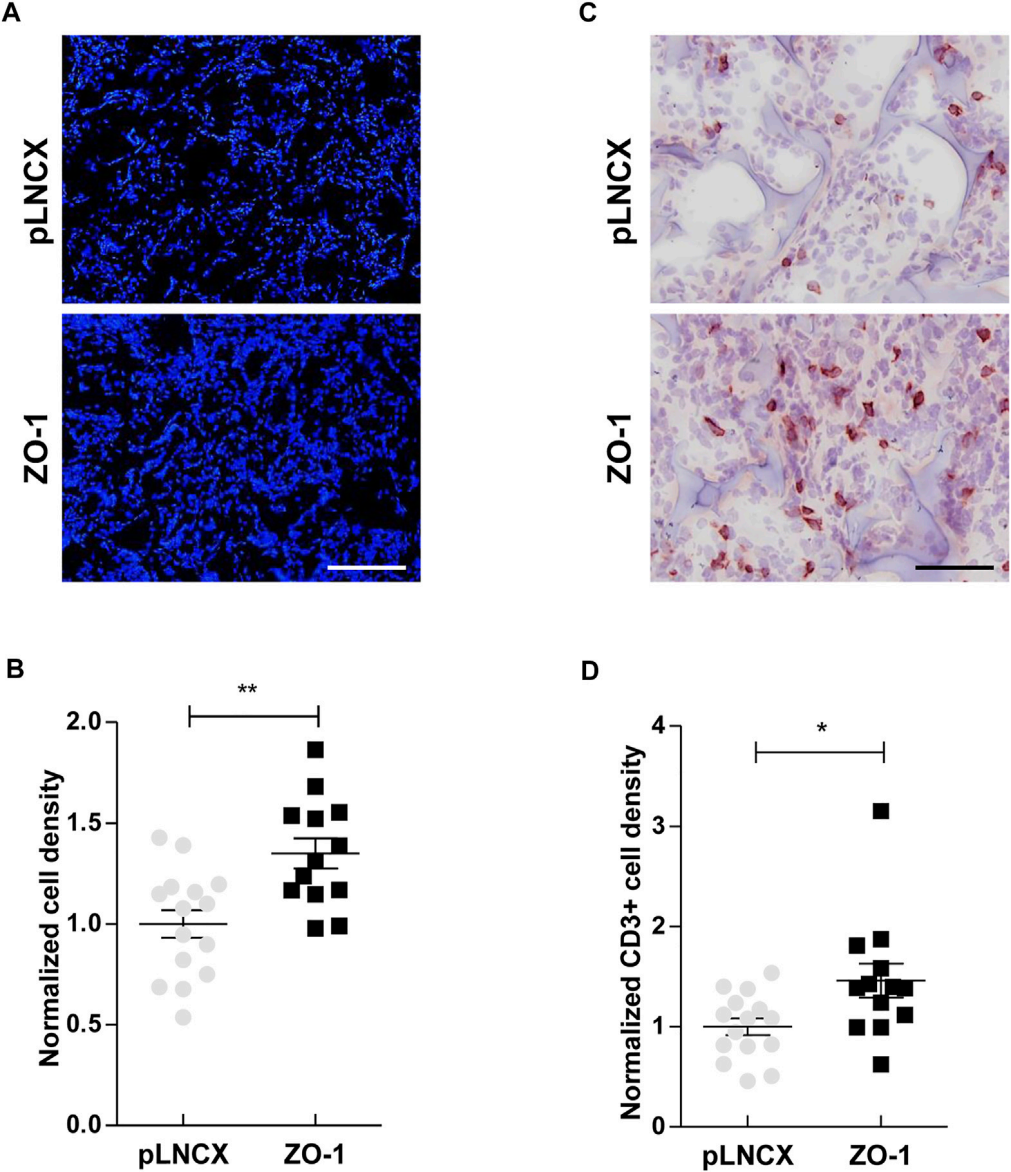
ZO-1 promotes an immune response *in vivo*. **(A)** DAPI staining on ear sections containing 21-days sponges soaked in conditioned medium of BEAS-2B cells transfected with ZO-1 expression vector (ZO-1) or the corresponding pLNCX control vector (pLNCX). Scale bar = 120 µm. **(B)** Cell density analysis performing by quantification of DAPI staining. **(C)** CD3 immunostaining on ear sections containing 21-days sponges soaked beforehand in conditioned medium of BEAS-2B cells transfected with ZO-1 cDNA or pLNCX empty vector. Scale bar = 80 µm. **(D)** T lymphocyte density analysis following quantification of CD3 labeling. Data are expressed as fold induction relative to the respective control conditions in three independent experiments. Means ± SEM; *n* = 15; *p < 0.05, **p < 0.01.

Aiming to explore this ZO-1/soluble factors/inflammatory infiltrate axis further in an *in vivo* context, we used a mouse ear sponge assay which is particularly adequate to analyze the impact of tumor-produced soluble factors on immune cell recruitment. For this assay, bio-compatible sponges were soaked in conditioned media of ZO-1-transfected BEAS-2B cells or control cells, and inserted subcutaneously in mouse ears for 3 weeks. An initial quantification of DAPI labeling permitted to quantify overall levels of cell infiltration in the sponges. Our results showed a larger cellular infiltration of sponges soaked in conditioned media from ZO-1-transfected BEAS-2B cells (1.35 ± 0.07-fold) (Figures 2A,B). Examining closer cellular infiltrates, we observed a higher recruitment of CD3^+^ T cells (1.46 ± 0.17-fold; Figures 2C,D) in the “ZO-1 sponges”. Similar results were obtained with SKBR3 cells, another cell model which maintains ZO-1 as a cyto-nuclear pool (1.90 ± 0.15 fold; Supplementary Figure S2). Taken together, these results suggest that the secretome of tumor cells with high cyto-nuclear levels of ZO-1 promotes recruitment of immune T cells.

### Cyto-Nuclear ZO-1 Expression Correlates With the Presence of an Immune Infiltrate in NSCLC

**FIGURE 3:**
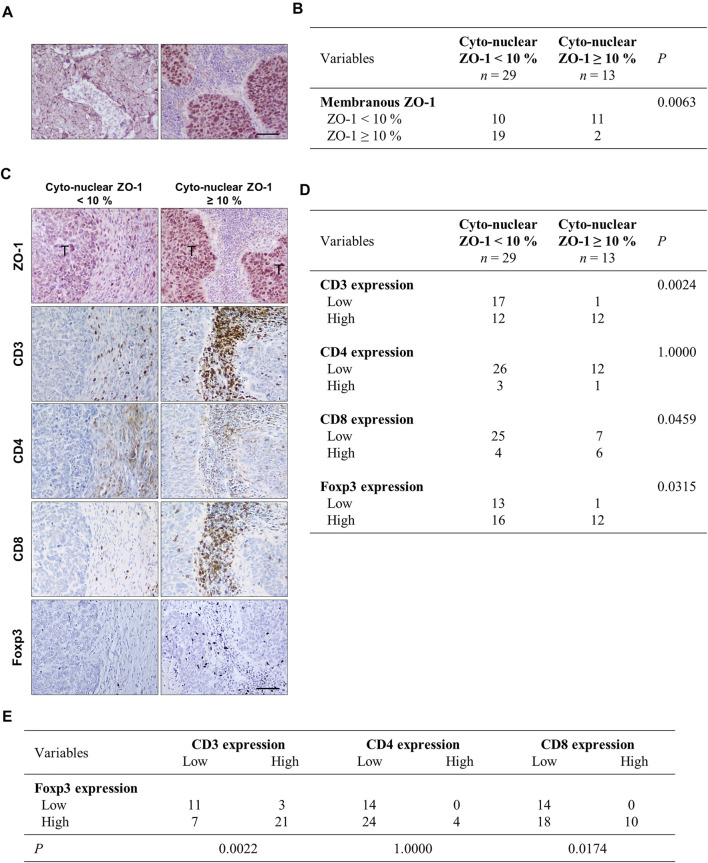
Cyto-nuclear localization of ZO-1 in tumor cells is associated with cytotoxic T cells and immunosuppressive cell recruitment in the tumor microenvironment in human lung cancers. **(A)** Immunohistochemistry of ZO-1 illustrating a membrane-associated ZO-1 staining **(left panel)** and a cyto-nuclear ZO-1 labeling **(right panel)** in human NSCL cancers. **(B)** Statistical analysis of associations between cyto-nuclear and membranous ZO-1 distribution. 42 human NSCL carcinomas were analyzed. Samples were scored for membrane-associated and cyto-nuclear ZO-1 distribution as detailed in the material and methods section. The numbers of cases falling in the different groups are given in the table. **(C)** Immunohistochemistry analysis showing ZO-1, CD3, CD4, CD8, and Foxp3 staining on serial sections of the NSCLC tumor samples. (T) indicates tumor clusters. Scale bar = 80 µm. **(D)** Statistical analysis of associations between cyto-nuclear localization of ZO-1 and Foxp3 and CD3, CD4, CD8 in NSCLC. **(E)** Statistical analysis of associations between Foxp3 and CD3, CD4, CD8 in the NSCLC samples.

Given these *in vitro* and *in vivo* results, we examined the potential relationship between ZO-1 localization in tumor cells and the inflammatory and immune infiltrate by immunostaining in human NSCLC samples. First, cancers were categorized according to ZO-1 staining pattern as displaying a low (<10% of tumor area) or a high (in ≥10% of tumor area) distribution of membrane-associated ZO-1, and a high (in ≥10% of tumor area) or low (<10% of tumor area) distribution of cyto-nuclear staining (an illustration of a ZO-1 membrane staining versus a cyto-nuclear staining is provided in Figure 3A). As shown in the scoring table (Figure 3B), cancers with high membrane-associated ZO-1 staining showed low cyto-nuclear ZO-1 labeling, and cancers with a high cyto-nuclear distribution of ZO-1 predominantly associated with a low ZO-1 membrane-associated score. Noticeably, cancers displaying a low ZO-1 membrane-associated score equally distributed between a group displaying a high cyto-nuclear ZO-1 distribution and a group displaying a low distribution of cyto-nuclear ZO-1, suggesting an overall weaker expression of ZO-1 in the latter. We next compared the immune infiltrate in the low and high cyto-nuclear ZO-1 distribution groups. We thus analyzed CD3 to identify T lymphocytes, and CD4 and CD8 to further differentiate T helper and cytotoxic T lymphocytes respectively (Figure 3C). We observed that a high cyto-nuclear staining of ZO-1 in lung tumor cells was associated with an increased density of T lymphocytes identified as a cytotoxic CD8^+^ T sub-population into tumor microenvironment (Figure 3D). Although these results are rather suggestive of an anti-tumor immune infiltrate in human NSCLC samples, we also evidenced a higher density of Foxp3^+^ immunosuppressive regulatory T cells in tumors with high cyto-nuclear expression of ZO-1 (Figure 3D). Interestingly, this higher density of Foxp3 regulatory T cells also correlated with a higher density of CD8^+^ cytotoxic T cells into the human lung tumor microenvironment (Figure 3E). Altogether, these results showed that the presence of cyto-nuclear ZO-1 in tumor cells correlated with an increased density of CD8^+^ and Foxp3^+^ immune cells in NCSLC. These *in vivo* data suggest that, even though more numerous, effector T cells might be inhibited by a higher recruitment of immunosuppressive T cells.

## Discussion

If many EMT-related studies have focused on deciphering intrinsic cellular changes in EMT cells, the role of EMT in modulating the interactions between epithelial tumor cells and host cells within the tumor microenvironment is becoming largely explored. In line with this stream of research, our results support the involvement of cyto-nuclear ZO-1, a subcellular localization generated by EMT process, in the increase of pro-inflammatory chemokine secretion and monocytic cell chemotaxis *in vitro*. *In vivo*, in an ear-sponge mouse model, we show an enhanced immune recruitment induced by ZO-1. In human lung cancers, we validate the presence of an increased immune infiltrate in the microenvironment of tumors with high cyto-nuclear ZO-1 content.

Thus, we here showed that the overexpression of ZO-1 in BEAS-2B invasive bronchial cells, which spontaneously maintain ZO-1 as a cyto-nuclear pool, induces the secretion of higher levels of several pro-inflammatory cytokines. Our results are in agreement with the work of Beutheu Youmba et al*.* who demonstrated that the subcellular redistribution of membrane ZO-1 in the cytoplasm of Caco-2 cells treated with methotrexate increases the expression of pro-inflammatory cytokines, such as IL-8 (Beutheu Youmba et al., 2012). Our data also complement works from Brysse et al*.* and Lesage et al*.* who established a correlation between the expression level of IL-8 and a cyto-nuclear localization of ZO-1 in breast and bronchial cell lines (Brysse et al., 2012; Lesage et al., 2017). In agreement with this reported influence of cyto-nuclear ZO-1 on tumor cell secretome, our *in vivo* sponge mouse model further demonstrated an increase of immune infiltrates in sponges soaked in conditioned media of cells overexpressing ZO-1 after 3 weeks of sponge implantation in the mouse ears. The establishment of a T immune response by ZO-1 in our *in vivo* murine sponge assay model may be related to our findings in human NSCLC establishing a correlation between cyto-nuclear expression of ZO-1 and higher density of CD3^+^ cells. Among this population, an important proportion appeared to be cytotoxic CD8^+^ T lymphocytes. Similarly, data from the literature also show significant infiltration of cytotoxic T cells in many cancers (Oelkrug and Ramage, 2014; Lou et al., 2016; Thompson et al., 2017). Among these studies, Lou et al*.* also showed an increase in T lymphocytes infiltrate in pulmonary adenocarcinomas (Lou et al., 2016). However, opposite observations have also been reported in the literature. Chae et al*.* for instance revealed an inverse correlation between EMT markers and the infiltration of T cells in NSCLC (Chae et al., 2018). In their work, ZO-1 was used as a marker for the loss of expression of epithelial proteins in EMT^+^ cells. This somehow contrasts with our study in which we show an inverse correlation between a low membranous ZO-1 expression and high cyto-nuclear ZO-1 expression in NSCLC, suggesting that the loss of expression of ZO-1 at the membrane can be relocated to another cell compartment. In addition, and partly conciliating these controversies, Romeo et al*.* proposed the existence of two potential scenarios on the behavior of EMT^+^ tumor cells to the recruitment of immune cells (Romeo et al., 2019): on one hand, EMT^+^ cells might induce immune infiltrate exclusion and on the other hand, EMT^+^ cells might exhaustively recruit deviated and/or immunosuppressive immune cells. Our results rather fit the second scenario. Indeed, although CD8^+^ cytotoxic T immune infiltrate was observed, Foxp3^+^ immunosuppressive regulatory T cells were also recruited in tumor areas displaying high cyto-nuclear ZO-1 that may potentially result in an overall immunosuppressive environment.

The mechanisms leading to ZO-1 relocalization to the cytoplasmic/nuclear compartments are still largely unclear. A loosening of cell-cell adhesion that coincides with the loss or downregulation of transmembrane proteins such as occludin, may directly contribute to facilitate ZO-1 subcellular relocalization. Additionally, ZO proteins harbor conserved nuclear localization and nuclear export motifs that may contribute to their nuclear shuttling. Further, through numerous protein/protein interactions domains, ZOs interact with various dual residency proteins thereby affecting their localization and functions (Bauer et al., 2010). If the dual localization of ZO-1 is now well documented (Gottardi et al., 1996; Reichert et al., 2000; Polette et al., 2007; Bauer et al., 2010), the specific functions endorsed by ZO-1 when distributed in the cyto/nuclear compartment remains elusive. Previous works from us and others nevertheless support a pro-tumoral function of cyto/nuclear ZO-1, stimulating EMT or the expression of MMP-14 (Reichert et al., 2000; Polette et al., 2007). Reinforcing these observations, our present data more particularly support that high levels of cyto/nuclear ZO-1 may upregulate pro-inflammatory molecule secretion and stimulate immune cell infiltration. It is important to note that inflammation has conversely been shown to weaken TJs in various inflammatory disease contexts (Wang et al., 2016; Bhat et al., 2019) and is a known potent inducer of EMT in cancer contexts (Suarez-Carmona et al., 2017), supporting the existence of crucial regulatory loops.

In conclusion, our results come to strengthen existing literature data that demonstrate an implication of EMT in regulating immune cell recruitment in the tumor microenvironment. They more particularly provide new insights into the role of ZO-1 in such mechanisms.

## Data Availability

The raw data supporting the conclusion of this article will be made available by the authors, without undue reservation.

## Abbreviations

ADC, Adenocarcinoma; CAF, Cancer-Associated Fibroblast; EMT, Epithelial-mesenchymal Transition; GK, Guanylate Kinase homologous; GM-CSF, Granulocyte-Macrophage Colony-Stimulating Factor; GROα, Growth-Regulated Oncogene-α; ICAM-1, InterCellular Adhesion Molecule-1; IL, InterLeukine; MAGUK, Membrane-Associated GUanylate Kinase homolog; MCP-1, Monocyte Chemoattractant Protein-1; MMP, Matrix MetalloProteinase; NES, Nuclear Export Signal; NLS, Nuclear Localization Signal; NSCLC, Non-Small Cell Lung Cancer; PDZ, PSD95/DLG/ZO-1; SCC, Squamous Cell Carcinoma; SH3, Src Homology 3; TAM, Tumor-Associated Macrophage; TGF-β, Transforming Growth Factor-beta; TIL, Tumor-Infiltrating Lymphocyte; TJ, Tight Junction; TNF-α, Tumor Necrosis Factor-alpha; VEGF, Vascular Endothelial Growth Factor; ZO, Zonula Occludens.
