# Supplementary material for: ZO-1 Intracellular Localization Organizes Immune Response in Non-Small Cell Lung Cancer
Source: Front Cell Dev Biol. 2021 Dec 6;9:749364. doi: 10.3389/fcell.2021.749364 (PMC8685499; doi:10.3389/fcell.2021.749364)
Supplement: Supplementary file 1 [file DataSheet1.docx]

Supplementary Material

**pLNCX**


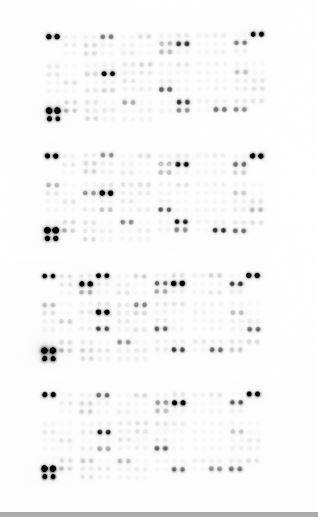

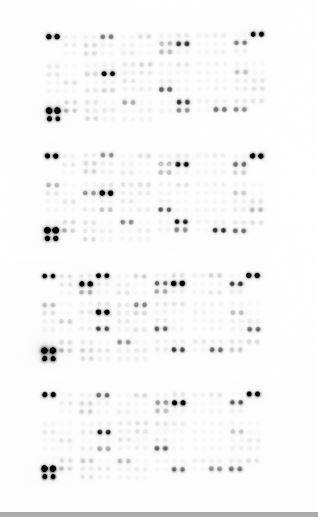


**ZO-1**

(1)

(3)

(6)

(2)

(2)

(5)

(1)

(3)

(6)

(5)

(4)

(4)

**Supplementary Figure 1.** Human cytokine-array membrane used with conditioned medium from BEAS-2B cells transfected with ZO-1 expression vector (*bottom*) or the corresponding pLNCX control vector (*top*). (1) = Groα, (2) = GM-CSF, (3) = IL-6, (4) = MMP-9, (5) = ICAM-1, (6) = IL-8.

**
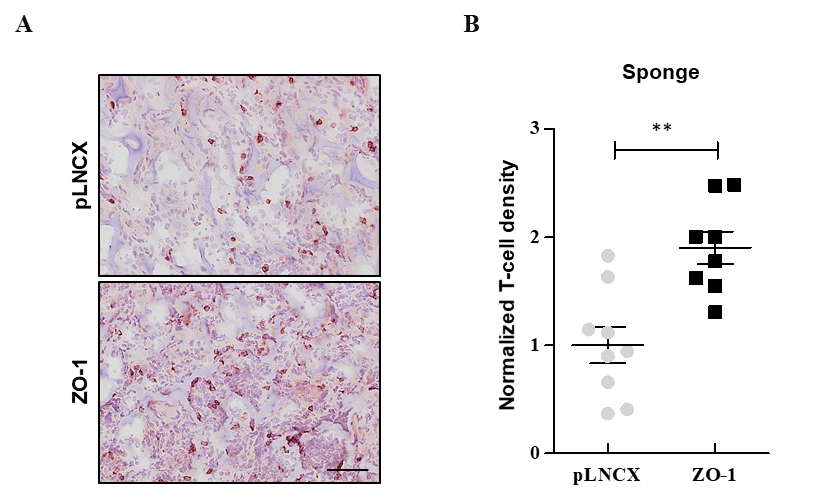
**

**Supplementary Figure S2.** Conditioned medium from ZO-1-tranfected SKBR3 cells promotes T cell recruitment. (A) CD3 immunostaining on ear sections containing 21-day sponges soaked beforehand in conditioned medium of SKBR3 cells transfected with ZO-1 cDNA (ZO-1) or pLNCX empty vector (pLNCX). (B) Lymphocyte T-cell density analysis by quantification of CD3 labelling. Scale bar = 80 µm. All data are normalized compared to the average of the respective control condition in 2 independent experiments. Means ± SEM; *n* = 10; ***P <* 0.01.

| **Variables** | **n = 42** |
| --- | --- |
| *Age (years)* | 64.5 ± 7.3 |
| *Sex* |  |
| Male | 33 (78.5%) |
| Female | 9 (21.5%) |
| *Histological type* |  |
| Adenocarcinomas | 21 (50.0%) |
| Squamous cell carcinoma | 21 (50.0%) |
| *Differentiation status* |  |
| Well | 12 (28.6%) |
| Mild | 11 (26.2%) |
| Poor | 19 (45.2%) |
| *Tumor size* |  |
| T1 | 15 (35.7%) |
| T2 | 19 (45.2%) |
| T3 | 7 (16.7%) |
| T4 | 1 (2.4%) |
| *Lymph node metastases* |  |
| N0 | 27 (64.3%) |
| N1 | 10 (23.8%) |
| N2 | 5 (11.9%) |
| *Tumor stage* |  |
| IA | 13 (31.0%) |
| IB | 9 (21.4%) |
| IIA | 8 (19.1%) |
| IIB | 3 (7.1%) |
| IIIA | 9 (21.4%) |

**Supplementary Table 1.** Characteristics of the population cohort studied by immunohistochemistry

**Supplementary Table S2.** List of antibodies used for immunohistochemistry

| **Antibody** | **Clone** | | **Source** | |
| --- | --- | --- | --- | --- |
| Anti-human ZO-1 | | ZO-1-1A12 | | Thermo Fischer Scientific |
| Anti-human CD3 | | - | | Dako |
| Anti-human CD4 | | RBT-CD4 | | Diagomics |
| Anti-human CD8 | | CD8-C8/144B | | Dako |
| Anti-human Foxp3 | | Foxp3-236A/E7 | | eBioscience |
